# One-year Mortality of Cancer Patients with an Unplanned ICU Admission: A Cohort Analysis Between 2008 and 2017 in the Netherlands

**DOI:** 10.1177/08850666211054369

**Published:** 2021-11-17

**Authors:** Esther N. van der Zee, Fabian Termorshuizen, Dominique D. Benoit, Nicolette F. de Keizer, Jan Bakker, Erwin J.O. Kompanje, Wim J.R. Rietdijk, Jelle L. Epker

**Affiliations:** 1 6993 Erasmus University Medical Center, Rotterdam, the Netherlands; 2 National Intensive Care Evaluation (NICE) foundation, Amsterdam, the Netherlands; 3 Amsterdam University Medical Center, Amsterdam Public Health research institute, 213752 University of Amsterdam, Amsterdam, the Netherlands; 4 60200 Ghent University Hospital, Ghent, Belgium; 5 5894 New York University, New York, USA; 6 21611 Columbia University Medical Center, New York, USA; 7 28033 Pontificia Universidad Católica de Chile, Santiago, Chile

**Keywords:** intensive care unit, critical care, malignancy, cancer, neoplasm, ICU admission, mortality, survival, time trend, ICU triage

## Abstract

**Introduction:** A decrease in short-term mortality of critically ill cancer patients with an unplanned intensive care unit (ICU) admission has been described. Few studies describe a change over time of 1-year mortality. Therefore, we examined the 1-year mortality of cancer patients (hematological or solid) with an unplanned ICU admission and we described whether the mortality changed over time. **Methods:** We used the National Intensive Care Evaluation (NICE) registry and extracted all patients with an unplanned ICU admission in the Netherlands between 2008 and 2017. The primary outcome was 1-year mortality, analyzed with a mixed-effects Cox proportional hazard regression. We compared the 1-year mortality of cancer patients to that of patients without cancer. Furthermore, we examined changes in mortality over the study period. **Results:** We included 470,305 patients: 10,401 with hematological cancer, 35,920 with solid cancer, and 423,984 without cancer. The 1-year mortality rates were 60.1%, 46.2%, and 28.3% respectively (*P*< .01). Approximately 30% of the cancer patients surviving their hospital admission died within 1 year, this was 12% in patients without cancer. In hematological patients, 1-year mortality decreased between 2008 and 2011, after which it stabilized. In solid cancer patients, inspection showed neither an increasing nor decreasing trend over the inclusion period. For patients without cancer, 1-year mortality decreased between 2008 and 2013, after which it stabilized. A clear decrease in hospital mortality was seen within all three groups. **Conclusion:** The 1-year mortality of cancer patients with an unplanned ICU admission (hematological and solid) was higher than that of patients without cancer. About one-third of the cancer patients surviving their hospital admission died within 1 year after ICU admission. We found a decrease in 1-year mortality until 2011 in hematology patients and no decrease in solid cancer patients. Our results suggest that for many cancer patients, an unplanned ICU admission is still a way to recover from critical illness, and it does not necessarily lead to success in long-term survival. The underlying type of malignancy is an important factor for long-term outcomes in patients recovering from critical illness.

## Introduction

Worldwide, approximately 40% of all people are diagnosed with cancer during their lifetime.^1^ In general, malignancies remain a major cause of death in Europe and North America.^2,3^ Fortunately, due to early detection and improved cancer treatment, long-term mortality of cancer patients has decreased over the past decades.^1,4,5^ However, the probability of life-threatening events related to therapy requiring intensive care has increased.^6^ Therefore, intensivists worldwide are increasingly confronted with cancer patients.^7,8^

Until the early 2000s, patients with advanced malignancy were generally perceived as ineligible for intensive care treatment due to the unfavorable outcomes.^9-11^ Nowadays, around 10%-20% of the patients admitted to an intensive care unit (ICU) have cancer.^12–14^ Several studies show that ICU and hospital mortality of cancer patients has decreased over the years.^9,14–19^ In particular, a good prognosis in planned postsurgical cancer patients has been reported.^8,15,18,20^ Therefore, these patients are generally accepted to the ICU for postoperative care.

In contrast, studies reporting recent data regarding long-term mortality of cancer patients following unplanned ICU admission are scarce, especially studies describing a change over time of 1-year mortality. Recent data will complement previous literature,^18–24^ and is relevant for two reasons. First, in an acute setting, ICU physicians may be confronted with critically ill cancer patients. An enhanced understanding of outcomes in this patient group may lead to improved treatment decisions of hematologists, oncologists, and ICU physicians. Second, updated data may help to create realistic expectations toward patients and their relatives regarding ICU treatment and short-term and long-term mortality.

Therefore, the aim of our study was to determine whether the observed decrease in short-term mortality of cancer patients with an unplanned ICU admission is associated with a decrease in 1-year mortality. We assessed 1-year mortality of patients with a malignancy (hematological or a solid cancer) and without malignancy with an unplanned admission to the ICU. Subsequently, we assessed whether there was a trend in 1-year mortality over the study period.

## Methods

### Patient Data

The National Intensive Care Evaluation (NICE) registry was used to identify all adult patients with an unplanned admission to ICUs in the Netherlands from 2008 to 2017. This registry contains data of patients from 85% of the Dutch ICUs in 2008 to 100% of the Dutch ICUs in 2017.^25^ An unplanned ICU admission was defined as an admission due to an acute medical condition or an admission following scheduled surgery with intra-operative complications requiring ICU admission.

The NICE registry contains data on ICU admission (planned or unplanned), demographics, physiological and diagnostic data, ICU characteristics, and ICU and hospital mortality of patients. We linked a national administrative insurance claims database (Vektis) to the NICE registry, to determine the 1-year mortality as previously reported.^26,27^ Health insurance is obligatory for all Dutch citizens and 99% have private healthcare insurance.^28^ The Vektis databases^29^ contain reimbursement data on all medical treatments paid for by Dutch insurance companies, as well as demographic information, such as date of birth, gender, and a proxy for date of death for all registered inhabitants of the Netherlands.

We stratified the patients into three cohorts: patients with hematological cancer, patients with a solid malignancy, and patients without cancer.

Patients with a hematological cancer were defined using the following three criteria: (i) patients referred from the hematological ward, (ii) patients with a hematological malignancy (e.g. leukemia or lymphoma) as admission diagnosis, or (iii) patients with “hematological malignancy” as comorbidity.

Patients with a solid malignancy were defined using the following three criteria: (i) patients referred from the oncology ward, (ii) patients with a solid malignancy as admission diagnosis, or (iii) patients with a comorbid condition “metastasized neoplasm”.

The ICU patients without cancer were defined as all patients not included in the cohorts mentioned above.

All patients with a planned ICU admission were excluded. If patients were admitted to the ICU multiple times during the same hospitalization, only the first ICU admission was included in the analysis. Patients who underwent elective surgery and who subsequently had an unplanned ICU admission due to complications (e.g. after major bleeding during surgery) were included in this study as unplanned admissions.

### Baseline Characteristics and ICU Admission Characteristics

A complete list of the characteristics and definitions can be found in Supplementary Table 1.

### Primary and Secondary Outcomes

The primary outcome was 1-year mortality. Secondary outcomes were ICU and hospital mortality.

### Ethics

The Medical Ethics Review Committee Erasmus Medical Center Rotterdam (decision number MEC-2019-0779) approved this study. Data are pseudonymized stored in the NICE registry and analyses were performed on an encrypted dataset. Because of the retrospective nature of the study and the usage of anonymous data, no additional informed consent was necessary. All procedures are in line with the General Data Protection Regulation (GDPR, May 2018) and law and regulations of the Netherlands.

### Statistical Analysis

Baseline and ICU admission characteristics of the study population were reported as count (percentage) for categorical variables or as mean (standard deviation) for continuous variables. Differences between the three cohorts were analyzed with the chi-square test for categorical variables and the Wilcoxon test for continuous variables.

The 1-year mortality rates are reported as count and percentages for the three cohorts separately, differences were calculated with the chi-square test. We analyzed the 1-year mortality trend over the inclusion period using a mixed-effects Cox proportional hazard model with the calendar year 2008 to 2017 as an independent variable, using dummies with 2008 as reference. We used hospital as a random intercept. The models were adjusted for Acute Physiology and Chronic Health Evaluation (APACHE) IV mortality probability. Since the APACHE IV mortality probability also corrects for admission reasons besides age, physiology, and underlying illness, we chose APACHE IV mortality probability instead of the APACHE IV score. For this analysis, we presented hazard ratios (HRs) and 95% confidence intervals (95% CIs). Further, we used a post-estimation Wald test to examine whether there were statistically significant differences in 1-year mortality over the inclusion period. This test examines the effect of including year dummies on mortality; however, it does not provide any information on whether there is a decreasing or increasing trend over the inclusion period. Therefore, we plotted the HRs and 95% CIs in a graph. We performed a visual inspection on this graph, in which we compared HRs per calendar year to HRs in earlier years over the inclusion period, in order to determine whether a decreasing or increasing trend could be found. Moreover, when using a post-estimation Wald test, statistically significant differences and *P*-values refer to sample variation. Since we included all patients with an unplanned ICU admission to the Dutch ICUs during the study period, we did not use a sample. The post-estimation Wald test is therefore subordinate to the visual inspection.

The secondary outcomes were analyzed using a similar strategy, with the exception that a mixed-effects binary logistic regression analysis, with the calendar year (2008-2017) as an independent variable, was used. This model was adjusted for APACHE IV mortality probability and included a random intercept for the hospital. For this analysis, we present the odds ratios (OR) with their 95% CI. All analyses were performed using R-studio 3.6.1. A *P* value of < .05 was considered statistically significant.

## Results

### Baseline and Intensive Care Unit Admission Characteristics

During the study period, 470,305 patients had an unplanned ICU admission, of these 10,401 (2.2%) patients had hematological cancer, 35,920 (7.6%) patients had a solid malignancy, and 423,984 (90.2%) patients had no cancer. In Supplementary Table 2, an overview of missing values for all cohorts is reported.

Baseline and ICU admission characteristics of the three cohorts are shown in Table 1. All differences between the cohorts were statistically significant (*P* < .05). The APACHE IV score was significantly higher in patients with hematological cancer compared to patients with a solid malignancy and patients without cancer, where there was no difference between the solid cancer and no-cancer patients (median 86 vs 59 and 57, respectively, *P* < .01). Within 24 h of ICU admission, 20.7% of the patients with hematological cancer had developed acute kidney failure (AKI), versus 10.2% in patients with a solid malignancy, and 11.1% in patients without cancer. Mechanical ventilation was used in 50.3% of the patients with hematological cancer, in 36.5% of the patients with solid cancer, and in 45.4% of patients without cancer. Vasoactive drugs were used in 47.2% of patients with hematological cancer, in 36.7% in patients with a solid malignancy, and in 34.6% of patients without a malignancy.

**Table 1.** Baseline and ICU Admission Characteristics.

	Hematological malignancy (n = 10,401)	Solid malignancy (n = 35,920)	Without malignancy (n = 423,984)	
Age (years)	63.9 (13.9)	67.5 (12.0)	62.3 (17.3)	
Gender (male)	6495 (62.5%)	20,805 (57.9%)	238,323 (56.2%)	
BMI	25.2 (4.8)	25.8 (5.1)	26.4 (5.9)	
APACHE IV score	86 [68-109]	59 [43-81]	57 [38-81]	
Comorbidity			
COPD and respiratory insufficiency	1405 (13.9%)	5687 (15.8%)	75,214 (17.7%)	
Renal insufficiency and dialysis	858 (8.2%)	1651 (4.6%)	27,699 (6.5%)	
Liver cirrhosis	110 (1.1%)	337 (0.9%)	7806 (1.8%)	
Cardiovascular insufficiency	328 (3.2%)	1050 (2.9%)	21,645 (5.1%)	
Immunological insufficiency	6210 (59.7%)	6121 (17.0%)	23,621 (5.6%)	
Underlying type hematological malignancy			
Leukemia	1342 (12.9%)			
Hodgkin lymphoma	118 (1.1%)			
Non-Hodgkin lymphoma	813 (7.8%)			
Bone marrow transplant	57 (0.5%)			
Hematological malignancy, not specified	8071 (77.6%)			
Specification cohort 1			
Comorbidity: hematological malignancy	6599 (63.4%)			
Referral specialism: hematology	284 (2.7%)			
Both comorbidity and referral specialism	802 (7.7%)			
APACHE IV diagnosis: hematological malignancy	2330 (22.4%)			
APACHE IV diagnosis: other	386 (3.7%)			
Underlying type solid malignancy			
Gastrointestinal malignancy		11,037 (30.7%)		
Pancreas malignancy		842 (2.3%)		
Renal/urogenital malignancy		2985 (8.3%)		
Neurological malignancy		2144 (6.0%)		
Respiratory malignancy		4696 (13.1%)		
Solid malignancy, not specified		14,216 (39.6%)		
Specification cohort 2			
Comorbidity: neoplasm		13,211 (36.8%)		
Referral specialism: oncology		183 (0.5%)		
Both comorbidity and referral specialism		344 (1.0%)		
APACHE IV diagnosis: solid malignancy		21,704 (60.4%)		
APACHE IV diagnosis: other		478 (1.3%)		
Admission reason			
Respiratory failure	1038 (10.5%)	2062 (9.8%)	33,287 (7.9%)	
Sepsis	2454 (24.9%)	3484 (16.6%)	38,235 (9.0%)	
Pneumonia	2427 (24.6%)	2323 (11.0%)	40,097 (9.5%)	
Acute kidney injury/failure	163 (1.7%)	313 (1.5%)	4408 (1.0%)	
Complications of surgery	49 (0.5%)	730 (3.5%)	7375 (1.7%)	
Cardiac disease	582 (5.9%)	1289 (6.1%)	36,370 (8.6%)	
Cardiac arrest	351 (3.6%)	849 (4.0%)	27,074 (6.4%)	
Neurological	465 (4.7%)	1213 (5.8%)	38,350 (9.0%)	
Medication related	17 (0.2%)	81 (0.4%)	15,493 (3.7%)	
Gastrointestinal	885 (9.0%)	4388 (20.9%)	47,987 (11.3%)	
Metabolic/endocrine	176 (1.8%)	578 (2.7%)	11,362 (2.8%)	
Thromboembolism	146 (1.5%)	612 (2.9%)	11,548 (2.7%)	
Other	1111 (11.3%)	3078 (14.6%)	111,798 (26.4%)	
Admission type			
Medical	9212 (88.7%)	13,540 (38.0%)	296997 (71.4%)
Emergency surgery	888 (8.6%)	7435 (20.9%)	81295 (19.5%)
Elective surgery	280 (2.7%)	14,624 (41.1%)	37707 (9.1%)
Admitted from			
Emergency department	2243 (21.9%)	4286 (12.1%)	150,865 (36.6%)
Operation theatre	1019 (10.0%)	19,353 (54.5%)	101,675 (24.6%)
Ward	6101 (59.6%)	10,203 (28.7%)	122,253 (29.6%)
CCU/ICU	678 (6.6%)	950 (2.7%)	25,342 (6.1%)
Special/medium care	64 (0.6%)	299 (0.8%)	2748 (0.7%)
Home	78 (0.8%)	191 (0.5%)	5469 (1.3%)
Other	49 (0.5%)	231 (0.7%)	4409 (1.1%)
Diagnoses at admission			
CPR	476 (4.6%)	1130 (3.1%)	30,616 (7.2%)
Gastrointestinal bleeding	365 (3.5%)	1105 (3.1%)	12,960 (3.1%)
Cardiovascular	1461 (14.1%)	3290 (9.2%)	57,336 (13.5%)
Neurological	368 (3.5%)	1950 (5.4%)	12,960 (3.1%)
Diabetes	1318 (12.7%)	5103 (14.2%)	68,826 (16.2%)
Diagnoses within 24 h of ICU admission			
Acute renal failure	2155 (20.7%)	3663 (10.2%)	47,142 (11.1%)
Mechanical ventilation	5236 (50.3%)	13,105 (36.5%)	19,2411 (45.4%)
Confirmed infection	4116 (39.6%)	6639 (18.5%)	83,919 (19.8%)
Vasoactive drugs	4913 (47.2%)	13,186 (36.7%)	146,767 (34.6%)

Data are displayed as mean (standard deviation) for continuous and count (percentages) for categorical variables, all *P* values was highly significant with a *P* < 0.01.

Abbreviations: APACHE, Acute Physiology and Chronic Health Evaluation; BMI, body mass index; CCU, coronary care unit; CPR, cardiopulmonary resuscitation; COPD, chronic obstructive pulmonary disorder; ICU, intensive care unit.

### Primary Outcome

**Figure 1.**
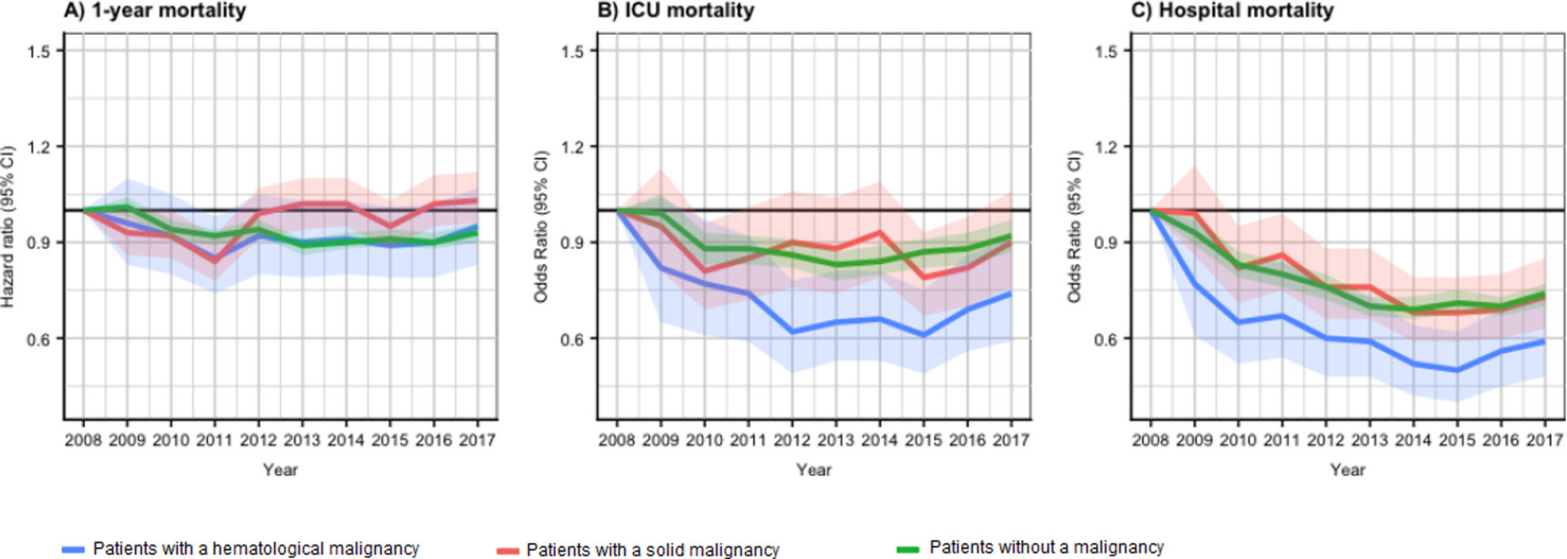
(A) Shows the trend of hazard ratios (HRs) for 1-year mortality over the inclusion period for the three cohorts (hematological patients, *P*= .58; solid malignancy, *P* < .01; patients without a malignancy, *P* < .01). (B) Shows the trend of odds ratios (OR) for ICU mortality over the inclusion period for the three cohorts (hematological patients, *P* < .01; solid malignancy, *P* = .11; patients without a malignancy, *P* < .01). (C) Shows the trend of OR for hospital mortality over the inclusion period for the three cohorts (hematological patients, *P* < .01; solid malignancy, *P* < .01; patients without a malignancy, *P* < .01). *P* values are based on a post-estimation Wald test. The actual hazard and odds ratios are available on request from the corresponding author.

Abbreviation: ICU, intensive care unit.

The 1-year mortality rate of patients with hematological cancer, a solid malignancy, and without cancer was 60.1%, 46.2%, and 28.3%, respectively (*P* < .01, Table 2). Figure 1A shows the trend of HRs for 1-year mortality over the study period for the three cohorts. For hematological patients, visual inspection of the 1-year mortality graph showed a decreasing trend until 2011, after which the mortality stabilized. The post-estimation Wald test showed no differences in mortality per year over the inclusion period (*P* = .58). In contrast, for patients with a solid malignancy, the post-estimation Wald test showed a difference in 1-year mortality per year over the inclusion period (*P* < .01). Visual inspection suggested this difference was mainly due to significantly lower HR in 2011, with a wide variation between HRs over other years, in neither an increasing nor decreasing trend. For patients without cancer, visual inspection of the 1-year survival graph suggested a decreasing trend in HRs until 2013, after which the mortality stabilized. The post-estimation Wald test found a difference in 1-year mortality over the study period (*P* < .01).

**Table 2.** Overview of Mortality Rates and ICU/Hospital Length of Stay.

	Hematological malignancy (n = 10,401)	Solid malignancy (n = 35,920)	Without malignancy (n = 423,984)
ICU mortality	2969 (28.6%)	4890 (13.6%)	52,864 (12.5%)
Hospital mortality	3724 (37.9%)	6916 (20.0%)	65,026 (16.4%)
1-year mortality	5916 (60.1%)	15,606 (46.2%)	111,328 (28.3%)
ICU length of stay (days)	2.4 [0.9-6.2]	1.1 [0.8-2.9]	1.6 [0.7-3.8]
Hospital length of stay after ICU (days)	10.3 [4.3-20.5]	8.6 [4.4-15.4]	7.5 [3.2-15.4]

All *P*-values assessing statistical differences between cohorts were highly significant with a *P* < .01.

Patients who were lost to follow-up are not included in this table.

Abbreviation: ICU, intensive care unit.

### Secondary Outcomes

ICU mortality of hematological patients was 28.6%, of patients with a solid malignancy 13.6%, and of patients without cancer 12.5% (*P* < .01, Table 2). Figure 1B shows the trend of OR for ICU mortality over the study period for the three cohorts.

For patients with a hematological malignancy visual inspection suggested a decrease in ORs until 2012, with a slight increase from 2015 to 2017. The Wald test found a difference in ICU mortality over the inclusion period (*P* <.01). For patients with a solid malignancy, visual inspection suggested a varying trend in HRs per year, with no clear decrease or increase. The Wald test found no difference in ICU mortality over the inclusion period (*P* = .11). For patients without cancer visual inspection suggested a decreasing trend until 2013, after which the mortality stabilized. The Wald test found a difference in ICU mortality over the inclusion period (*P* < .01).

The hospital mortality of hematological patients was 37.9%, of patients with a solid malignancy 20.0%, and of patients without cancer 16.4%, (*P* < .01). Figure 1C shows the trend of OR for hospital mortality over the inclusion period for the three cohorts. We found with a visual inspection a decrease in hospital mortality for each cohort over the inclusion period. The post-estimation Wald test suggested a difference for each of the three cohorts as well (*P* < .05).

### Additional Post Hoc Observations

Three additional factors, relevant for the interpretation of our results, were also analyzed. First, when patients with cancer (either hematological or solid) survived hospital admission, approximately one-third died (30%) within the year following that ICU admission. In patients without cancer, this number was lower (12%).

Second, visual inspection of the APACHE IV probability of mortality over the study period suggested no major changes in illness severity at admission over the inclusion period for each cohort. The post-estimation Wald test showed differences in APACHE IV probability of mortality between calendar years over the inclusion period for all three cohorts (p < .01). The APACHE IV probability of mortality for each cohort is presented in Supplementary Figure 1).

Third, the proportion of patients in the three cohorts was similar in each year during the study period. Patients with hematological malignancy represented 2% to 3%, patients with a solid malignancy 7% to 8%, and on average 90% of the admitted patients had no cancer (Supplementary Table 3).

## Discussion

In this study, we showed that in the period of 2008 to 2017, cancer patients (either hematological or solid) with unplanned ICU admission had a significantly higher 1-year mortality compared to patients without cancer with an unplanned ICU admission. In patients with hematological cancer, we found a decrease in 1-year mortality from 2008 to 2011, after which mortality stabilized. In patients with solid cancer, no change was seen. We found evidence for an overall decreasing trend in hospital mortality of critically ill cancer patients. Apparently, for many cancer patients, ICU admission is still a bridge to recover from their critical illness. It however does not necessarily lead to success in long-term survival, the underlying type of malignancy or its treatment is an important factor for long-term outcomes in patients recovering from critical illness.

In contrast to the decrease in long-term mortality over the past decades of cancer patients in general,^1,4,5^^)^ in this cohort of patients with an unplanned ICU admission, we found only a small decrease in 1-year mortality in hematological patients and no decrease in solid cancer patients. The variety in underlying types of malignancy and treatment options for those different malignancies might explain the slight or absent decrease in 1-year mortality of patients with cancer and an unplanned ICU admission.^19^ Interestingly, a recent study reporting mortality trends of septic shock patients showed a decrease in mortality between 2009 and 2011 after which it stabilized.^30^ In our study population, a considerable part (particularly in those with a hematological malignancy) was admitted with sepsis or infection to the ICU. The absence of new treatments since 2011 for sepsis and septic shock might explain the absence of a decrease in mortality after 2011.

Complementary to other literature, our study presents long-term mortality data from a nationwide registry in cancer patients with an unplanned ICU admission.^18–24^ Differences in case mix between our study and previous literature make direct comparison difficult. For example, a large study performed in the United States with data from 2002 to 2011^19^ reported a decrease in 1-year mortality of patients with hematological cancer and solid cancers. However, this study included both planned and unplanned ICU admissions. Interestingly, the final year of their database (2011) was similar to the year in which in our database, the decrease in 1-year mortality of hematological patients ended. Other studies regarding long-term mortality do not report trends over time. For example, Puxty et al.^18^ compared mortality rates of surgical patients with and without cancer admitted to the ICU. They reported a lower 1-year mortality when compared to our study, however, the majority of their patients had a planned ICU admission. Further, a study from Belgium^20^ showed comparable mortality for patients with hematological cancers to our study, while the 1-year mortality of patients with a solid malignancy was lower than in our study. Again, the study of Oeyen et al. included both planned and unplanned ICU patients. Two studies (Ehooman et al.^24^ and Azoulay et al.^31^) included only hematological patients. ICU, hospital, and 1-year mortality rates of their patients were compared to that of the hematological patients in our study. Finally, the 1-year mortality of cancer patients in our study was lower than that of studies with smaller sample sizes from Spain, Austria, and Switzerland.^21–23^ Apart from sample size, there is only a small difference in case-mix between our study and these three studies. Although no decrease in 1-year mortality was seen in our study, the decrease in mortality when compared to these studies might be a reason for cautious optimism regarding long-term mortality.

As a secondary outcome, we found a decrease in hospital mortality over the inclusion period for all three cohorts. These results are in line with several other studies that showed a decreasing trend in hospital mortality of cancer patients.^9,14–19^ Relevant to note is that when cancer patients (either hematological or solid) survived the hospital admission, approximately one-third died within the year following the ICU admission. In patients without cancer, this number was considerably lower (12%).

One of the strengths of our study is that it adds to current literature. Especially, the fact that the underlying type of malignancy or its treatment may be an important factor for long-term outcomes in patients recovering from critical illness. A large recent study^32^ showed similar results. Among all diagnostic subgroups of the ICU population included in that study, patients with nonsurgical cancer had the lowest cumulative 1-year survival. A possible explanation could be that a considerable part of the deaths within one year in the cancer cohorts is directly or indirectly related to the underlying malignancy or its treatment. This may explain the clear improvement in short-term mortality of cancer patients, while no clear improvement in long-term mortality was seen. A possible and not unlikely explanation for the decrease in hospital mortality and high 1-year mortality may be the physician's decision to discharge patients to a nursing home or hospice for the terminal phase, leading to a spurious reduction of mortality risk when using hospital death as an outcome.^33,34^ Unfortunately, we have no data available in the NICE register to support this hypothesis. However, ICU admissions of patients are often determined by short-term survival benefit, and not by long-term prognosis.^35^ In addition, physicians do not always communicate adequately about the prognosis and benefits of treatments toward patients and relatives.^36–38^ Ideally, oncologists and hematologists discuss long-term outcomes after an ICU admission with patients and relatives well before an ICU admission, in order to manage expectations. All physicians should consider the benefits and burden of an ICU admission for each patient individually before ICU referral or admission.

### Limitations

For interpreting the study results, the following limitations should be considered.

First, our study is a general description of a heterogeneous population of critically ill cancer patients (both hematological malignancies and solid malignancies) with an unplanned ICU admission.

Second, we could not include the type and stage of the malignancy, the continuation of cancer treatment, and the performance status prior to ICU admission. Originally, the NICE registry is a quality of ICU care registry in the Netherlands, in which not all clinically relevant factors for cancer patients are registered. However, the impact on the outcomes of the current study might be limited as it is unlikely that cancer types and stages or performance status have significantly changed during the study period.^39^

Third, various reasons ICU admission exists in cancer patients. These factors were beyond the scope of this descriptive study and although relevant, cannot be reported.

Fourth, we performed a post-estimation Wald test and a visual inspection. To interpret our results, we used the visual inspection as the leading result. As described in the methods, the post-estimation Wald test is subordinate to the visual inspection.

Lastly, differences in treatment standards may occur for cancer patients across hospitals. However, by including a hospital random intercept in our models, we corrected for hospital variations. In addition, the actual numbers and proportions of cancer patients with an unplanned ICU admission were not different over the study period. This suggests that at least similar numbers of cancer patients were admitted without structural changes in admission policies.

### Future Research

Linking the NICE registry with the national cancer registry in the Netherlands may enrich the data with clinically relevant factors. Such enrichment may help to identify patients with a malignancy who are likely to benefit most from ICU admission.^6,7^

It would be helpful to establish more uniform inclusion criteria to enable a viable comparison between countries. To minimize other sources of heterogeneity, analysis on subgroups of cancer patients, such as septic and nonseptic patients, may be considered in future research.

## Conclusion

The 1-year mortality of cancer patients (hematological and solid) with an unplanned ICU admission is higher than that of patients without cancer. One-third of the cancer patients surviving ICU admission died within 1 year after ICU admission. We found a decrease in 1-year mortality until 2011 in hematology patients and no decrease in solid cancer patients. Our results suggest that in many cancer patients, an unplanned ICU admission is still a bridge to recover from critical illness, and it does not necessarily lead to success in long-term survival. The underlying type of malignancy is an important factor for long-term outcomes in patients recovering from critical illness.

## Abbreviations

AKI: acute kidney failure
APACHE: Acute Physiology and Chronic Health Evaluation
BMI: body mass index
CCU: coronary care unit
95% CIs: 95% confidence intervals
COPD: chronic obstructive pulmonary disease
CPR: cardiopulmonary resuscitation
HR: hazard ratio
ICU: intensive care unit
LOS: length of stay
NICE: National Intensive Care Evaluation
OR: odds ratio.

## Supplemental Material

sj-docx-1-jicm-10.1177_08850666211054369 - Supplemental material for One-year Mortality of Cancer Patients with an Unplanned ICU Admission: A Cohort Analysis Between 2008 and 2017 in the Netherlands Click here for additional data file. Supplemental material, sj-docx-1-jicm-10.1177_08850666211054369 for One-year Mortality of Cancer Patients with an Unplanned ICU Admission: A Cohort Analysis Between 2008 and 2017 in the Netherlands by Esther N. van der Zee, Fabian Termorshuizen, Dominique D. Benoit, Nicolette F. de Keizer, Jan Bakker, Erwin J.O. Kompanje, Wim J.R. Rietdijk and Jelle L. Epker in Journal of Intensive Care Medicine

sj-png-2-jicm-10.1177_08850666211054369 - Supplemental material for One-year Mortality of Cancer Patients with an Unplanned ICU Admission: A Cohort Analysis Between 2008 and 2017 in the Netherlands Click here for additional data file. Supplemental material, sj-png-2-jicm-10.1177_08850666211054369 for One-year Mortality of Cancer Patients with an Unplanned ICU Admission: A Cohort Analysis Between 2008 and 2017 in the Netherlands by Esther N. van der Zee, Fabian Termorshuizen, Dominique D. Benoit, Nicolette F. de Keizer, Jan Bakker, Erwin J.O. Kompanje, Wim J.R. Rietdijk and Jelle L. Epker in Journal of Intensive Care Medicine

sj-docx-3-jicm-10.1177_08850666211054369 - Supplemental material for One-year Mortality of Cancer Patients with an Unplanned ICU Admission: A Cohort Analysis Between 2008 and 2017 in the Netherlands Click here for additional data file. Supplemental material, sj-docx-3-jicm-10.1177_08850666211054369 for One-year Mortality of Cancer Patients with an Unplanned ICU Admission: A Cohort Analysis Between 2008 and 2017 in the Netherlands by Esther N. van der Zee, Fabian Termorshuizen, Dominique D. Benoit, Nicolette F. de Keizer, Jan Bakker, Erwin J.O. Kompanje, Wim J.R. Rietdijk and Jelle L. Epker in Journal of Intensive Care Medicine
