# Supplementary material for: One-year Mortality of Cancer Patients with an Unplanned ICU Admission: A Cohort Analysis Between 2008 and 2017 in the Netherlands
Source: J Intensive Care Med. 2021 Nov 17;37(9):1165–73. doi: 10.1177/08850666211054369 (PMC9396560; doi:10.1177/08850666211054369)

**Supplementary Material Table 1 List of variables and definitions of the baseline and ICU characteristics**

| Admission reason | Condition or critical illness leading to ICU admission |
| --- | --- |
| Admission reason: Acute kidney injury / failure | Included all Acute Physiology and Chronic Health Evaluation (APACHE) IV admission reasons related to acute kidney injury |
| Admission reason: Cardiac arrest | Included all Acute Physiology and Chronic Health Evaluation (APACHE) IV admission reasons related to cardiac arrest |
| Admission reason: Cardiac disease | Included all Acute Physiology and Chronic Health Evaluation (APACHE) IV admission reasons related to cardiac disease |
| Admission reason: complications of surgery | Included all Acute Physiology and Chronic Health Evaluation (APACHE) IV admission reasons related to surgery complications |
| Admission reason: Gastro-intestinal | Included all Acute Physiology and Chronic Health Evaluation (APACHE) IV admission reasons related to gastro-intestinal diseases |
| Admission reason: Medication related | Included all Acute Physiology and Chronic Health Evaluation (APACHE) IV admission reasons related to medication |
| Admission reason: Metabolic / endocrine | Included all Acute Physiology and Chronic Health Evaluation (APACHE) IV admission reasons related to metabolic / endocrine diseases |
| Admission reason: Neurological | Included all Acute Physiology and Chronic Health Evaluation (APACHE) IV admission reasons related to neurological diseases |
| Admission reason: Pneumonia | Included all Acute Physiology and Chronic Health Evaluation (APACHE) IV admission reasons related to pneumonia |
| Admission reason: Respiratory failure | Included all Acute Physiology and Chronic Health Evaluation (APACHE) IV admission reasons related to respiratory failure |
| Admission reason: Sepsis | Included all Acute Physiology and Chronic Health Evaluation (APACHE) IV admission reasons related to sepsis |
| Admission reason: Thromboembolism | Included all Acute Physiology and Chronic Health Evaluation (APACHE) IV admission reasons related to thromboembolism |
|  |  |
| Admission type: Elective surgery | ICU admissions after surgery at a time that suits both patient and surgeon or early surgery scheduled within 24 hours after OR indication |
| Admission type: Emergency surgery | ICU admission after immediate surgery where resuscitation, stabilisation and physiological optimization is performed directly prior to the surgical procedure or simultaneously with the surgical procedure |
| Admission type: Medical | All ICU admissions not directly from the operation room or the recovery. |
| APACHE IV score | Acute Physiology and Chronic Health Evaluation, the APACHEIII score used in the APACHE IV model  We used a non-calibrated APACHE IV score, as described in Zimmerman et al. (35). By using one APACHE IV model, the APACHE score is not influenced by time, and therefore by differences in medical care, leading to the same mortality prediction in patients with equal characteristics. |
|  |  |
| Comorbidity: Immunological insufficiency | Immunological insufficiency: Long term immunosuppressive therapy, or corticosteroid use (short term high and long term low dosages), or active chemotherapy or radiotherapy in the past year, or chemotherapy or radiotherapy for Hodgkin or non-Hodgkin lymphoma at any time for IC admission, or humoral or cellular deficiencies.  Or Aids: HIV-positive and clinical complications such as pneumocystis carinii pneumonia, Kaposi's sarcoma, lymphoma, tuberculosis of toxoplasma infection, or HIV-positive with CD4 < 200. |
| Comorbidity: Cardiovascular insufficiency | Angina or symptoms at rest or during minimal effort, such as dressing and personal hygiene (New York Heart Association class IV). |
| Comorbidity: COPD & respiratory insufficiency | COPD: Chronic condition in which pulmonary function deteriorates. The most important pathologies which fall into this category are chronic bronchitis, chronic bronchiolitis and emphysema  Respiratory insufficiency: chronic restrictive, obstructive or vascular conditions in the lungs resulting in very severe restriction of mobility, OR registered chronic hypoxia, secondary polycythaemia, severe pulmonary hypertension (PAP sys > 40 mm Hg), or respiratory dependence (e.g. O2-dependent active respiratory conditions, sarcoidosis, interstitial fibrosis, tuberculosis, chronic obstructive pulmonary diseases). |
| Comorbidity: liver cirrhosis | Positive biopsy and documented portal hypertension, or there have been previous periods of high gastrointestinal bleeding as a result of portal hypertension, or there have been previous periods of hepatic failure, coma or encephalopathy (before the current hospital admission). |
| Comorbidity: Renal insufficiency & dialysis | Raised serum creatinine > 177 umol/L (2.0 mg/dl) and renal insufficiency in the medical history (before the current hospital admission). Or if the patient has been receiving long-term haemodialysis or peritoneal dialysis prior to the current hospital admission. |
|  |  |
| Diagnoses at admission | A diagnosis present before the ICU admission |
| Diagnoses at admission: cardiovascular | If one of the next diagnoses is present in combination with hemodynamic instability in 24 hours before ICU admission: arrhythmia, paroxysmal tachycardia, atrial fibrillation with high ventricular response (≥120/minute), second-/third degree AV-block. Hemodynamic instability is defined as systolic blood pressure <90mmHg, heart frequency >140 beats per minute, or need for medical intervention such as inotropes/vasopressors, defibrillation, anti-arrhythmic medication or a pacemaker.  Or a myocardial infarction in the six months prior to IC admission. |
| Diagnoses at admission: CPR | CPR (heart massage) during the 24 hours before ICU admission. Defibrillation and / or cardioversion without a heart massage do not count as CPR. |
| Diagnoses at admission: Diabetes | Medication-dependent form of diabetes. This must have been diagnosed before the current IC admission. |
| Diagnoses at admission: Gastro-intestinal bleeding | Hematemesis, melena or a gastro-intestinal bleeding found by gastro-intestinal endoscopy in 24 hours before ICU admission |
| Diagnoses at admission: Neurological | Cerebral embolism, occlusion, bleeding, or infarction prior ICU admission or within 1 hour after ICU admission.  Or If an abscess, tumour, bleeding, or subdural contusion identified by CT/MRI and causes midline shift, obliteration/distortion of the cerebral ventricles, bleeding in cerebral ventricle/subarachnoid space, mass of >4cm or a contrast enhanced mass. If mass effect is present at least within 1 hour after ICU admission, a CT scan is not mandatory. |
| Solid malignancy | Patients from the oncology ward   Or solid malignancy (such as colorectal cancer or lung cancer) determined with Acute Physiology and Chronic Health Evaluation (APACHE) IV admission reasons   Or with a co-morbid condition ‘metastasized neoplasm' defined as 'Metastases which have been diagnosed by clinical examination or confirmed by a pathology report or if there is Stage IV cancer (not solely regional lymph nodes).' |
|  |  |
| Unplanned ICU admission | An ICU admission which was not known (not planned) before the actual ICU admission and could not have been delayed 12 hours without risk. |
|  |  |
| Within 24h ICU admission | Diagnoses or therapies used during the first 24 hours of ICU admission |
| Within 24h ICU admission: Acute renal failure | Renal replacement therapy (during first 24 hours of ICU stay), or Serum creatinine level greater than 1.5 mg/100 ml (or 133µmol/l) during the previous 24 hours, associated with oliguria. Oliguria is defined as urine production ≤150 ml over a period of 8 consecutive hours. This oliguria cannot be caused by a missing or tight-fitting urine catheter or due to incontinence. |
| Within 24h ICU admission: Confirmed infection | Confirmed infection upon admission or if infection is confirmed during the first 24 hours of IC treatment. Accepted confirmation of infection is culture results and Gram staining. Perioperative findings may qualify as evidence: for example faeces in the open peritoneal cavity with laparotomy scores ‘yes’. It is therefore also possible to score "yes" on basis of a very strong suspicion of infection in radiology (e.g. new infiltrate) in conjunction with the clinic (e.g. purulent sputum and fever). Laboratory confirmation (including verbal or fax confirmation) must be obtained during the first 24 hours of admission. If the results are received after this, a ‘no’ must be scored. If perioperative findings are used, the procedure must have taken place either immediately prior to ICU admission or during the first 24 hours of ICU treatment. If radiological or other imaging material is used, the evidence must be undisputed. |
| Within 24h ICU admission: Mechanical ventilation | Use of a ventilator at the moment of IC admission or immediately (within 15 minutes) thereafter. |
| Within 24h ICU admission: Vasoactive drugs | Continuous intravenous vasoactive medication for a minimum period of one hour during the first 24 hours of ICU admission. |

**Supplementary Material Table 2. Summary of percentage of missing values**

|  | **Haematological malignancy** | **Solid malignancy** | **Without**  **malignancy** |
| --- | --- | --- | --- |
|  | **(n=10,401)** | **(n=35,920)** | **(n=423,984)** |
| ICU | 4 (0.04%) | 4 (0.01%) | 146 (0.03%) |
| Hospital | 584 (5.6%) | 1,415 (3.9%) | 28,007 (6.6%) |
| 1-year | 564 (5.4%) | 2,116 (5.9%) | 29,922 (7.1%) |

**Supplementary Material Table 3. Count (percentage) of patients with a malignancy as compared to total number of patients in each year over the inclusion period.**

| Year | Haematological malignancy | Solid malignancy | Without malignancy |
| --- | --- | --- | --- |
| 2008 | 583 (2%) | 2,460 (8%) | 28,921 (90%) |
| 2009 | 810 (2%) | 3,331 (8%) | 36,779 (90%) |
| 2010 | 908 (2%) | 3,706 (8%) | 39,082 (90%) |
| 2011 | 990 (2%) | 3,794 (8%) | 40,757 (90%) |
| 2012 | 985 (2%) | 3,816 (8%) | 43,389 (90%) |
| 2013 | 1,130 (2%) | 3,705 (7%) | 44,827 (90%) |
| 2014 | 1,215 (2%) | 4,041 (8%) | 47,481 (90%) |
| 2015 | 1,233 (2%) | 3,961 (8%) | 48,116 (90%) |
| 2016 | 1,326 (3%) | 3,661 (8%) | 47,962 (91%) |
| 2017 | 1,221 (2%) | 3,445 (7%) | 46,670 (91%) |

**Supplementary Material Figure 1. Time trend APACHE IV score in cohort 1, 2 and 3**


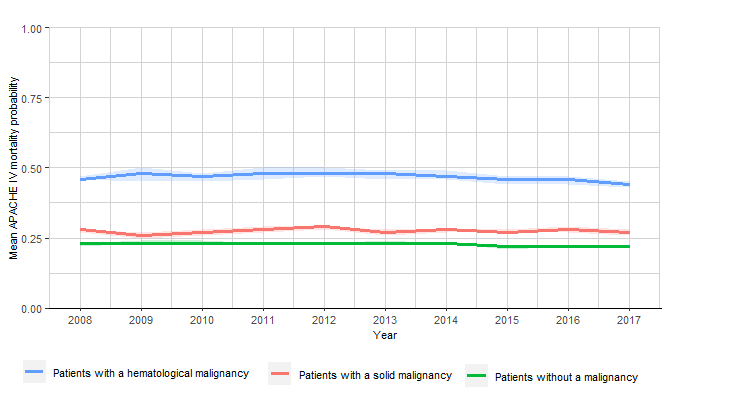

Supplement: sj-docx-1-jicm-10.1177_08850666211054369 - Supplemental material for One-year Mortality of Cancer Patients with an Unplanned ICU Admission: A Cohort Analysis Between 2008 and 2017 in the Netherlands [file sj-docx-1-jicm-10.1177_08850666211054369.docx]
