# Supplementary figures and images for: One-year Mortality of Cancer Patients with an Unplanned ICU Admission: A Cohort Analysis Between 2008 and 2017 in the Netherlands
Source: J Intensive Care Med. 2021 Nov 17;37(9):1165–73. doi: 10.1177/08850666211054369 (PMC9396560; doi:10.1177/08850666211054369)

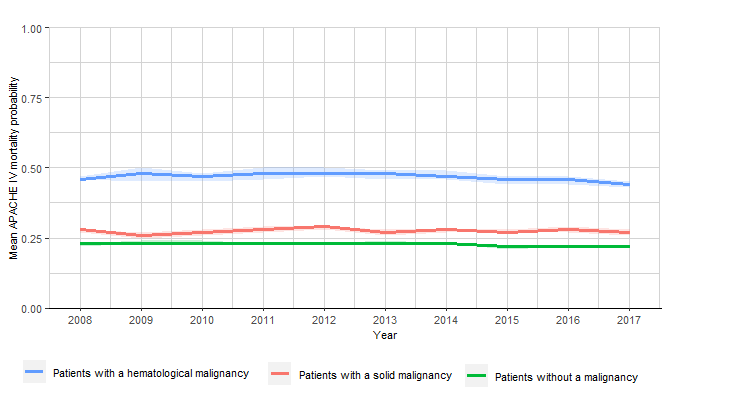

Supplement: sj-png-2-jicm-10.1177_08850666211054369 - Supplemental material for One-year Mortality of Cancer Patients with an Unplanned ICU Admission: A Cohort Analysis Between 2008 and 2017 in the Netherlands [file sj-png-2-jicm-10.1177_08850666211054369.png]
